# ﻿New taxa of *Plagiothecium* (Plagiotheciaceae) from Pakistan

**DOI:** 10.3897/phytokeys.236.109519

**Published:** 2023-11-23

**Authors:** Grzegorz J. Wolski, Aamir Shehzad Khan, Beata Paszko

**Affiliations:** 1 University of Lodz, Faculty of Biology and Environmental Protection, Department of Geobotany and Plant Ecology, Banacha 12/16, 90-237 Lodz, Poland University of Lodz Lodz Poland; 2 Peatland Ecology Research Group and Center of Nordic Studies, Department of Plant Sciences, Faculty of Agriculture and Food Sciences, Université Laval, Québec, QC G1V 0A6, Canada Université Laval Laval Canada; 3 Molecular Biogeography and Systematics Group, W. Szafer Institute of Botany, Polish Academy of Sciences, Lubicz 46, 31-512 Kraków, Poland W. Szafer Institute of Botany, Polish Academy of Sciences Kraków Poland

**Keywords:** Bryophyta, new taxa, Pakistan, Plagiotheciaceae, South Asia, taxonomy

## Abstract

A revision of specimens of *Plagiothecium* deposited in the herbarium of Pakistan Museum of Natural History (PMNH) collected during a Japanese lead project on Cryptogams in the Western Himalaya (Pakistan) shows that the material consists of five taxa. Of the studied samples, the most common taxa were from the *P.denticulatum* complex, including Plagiotheciumdenticulatumvar.obtusifolium, new to Pakistan. Examination of the rest of the collection showed that it consists of specimens with a unique combination of qualitative and quantitative characteristics of their gametophyte. For example, for small plants, with small asymmetrical, folded leaves, gradually tapering into long, acuminate, not denticulate apex, whose leaf cells are long and narrow, making the cell areolation tight, the name *Plagiotheciumfilifolium* is proposed. For other plants with large leaves, loosely arranged on the stem, concave, symmetrical to slightly asymmetrical, with denticulate apex and long decurrency composed of rectangular and spherical, inflated cells, the name *Plagiotheciumhiguchii* is proposed. However, within this material, specimens differ in terms of the length and width of the leaf cells and therefore, within this taxon, two varieties are distinguished: Plagiotheciumhiguchiivar.higuchii and Plagiotheciumhiguchiivar.brevicellum.

## ﻿Introduction

The northern part of Pakistan has a suitable climate and ambience for bryophytes, while the southern part of this country is nearly unexplored because it is far drier, having hot weather and some parts with arid and desert ecosystems. Thereby, due to difficult fieldwork, geographic and climatic conditions, this country is generally still poorly studied bryologically but mainly due to the lack of a resident bryologist. The scattered literature principally covers northern Pakistan and Western Himalaya (Asghar 1957; Froehlich 1963; Karczmarz 1980; Higuchi 1992; Nishimura et al. 1993a, b; Townsend 1993, 1994; Nishimura and Higuchi 2001; Higuchi and Nishimura 2003; Sollman 2008; Gruber and Peer 2012; Khan et al. 2020; Khan et al. 2021; Koponen et al. 2022).

The first checklist of mosses of Pakistan based on previous literature, and herbarium specimens, from Punjab and Khyber Pakhtunkhwa provinces was provided by Asghar (1957). Later, Higuchi and Nishimura (2003) presented a detailed list, primarily covering the moss flora of Northern part of this country and a few cities of Punjab province, which is currently considered the most valuable and authoritative source regarding the moss flora of this country. There is still a lot to be done to explore the moss flora because new studies have shown that there is a huge potential for new reports (Sollman 2008; Gruber and Peer 2012; Koponen and Higuchi 2020; Koponen et al. 2022).

Currently, the moss flora of Pakistan is represented by 319 species, with the largest family (Pottiaceae) consisting of 79 species (Sollman 2008; Gruber and Peer 2012; Koponen and Higuchi 2020; Koponen et al. 2022). According to investigations of the bryophytes by Higuchi and Nishimura (2003), nearly 45% of the species show a circumboreal distribution, with 32% belonging to an Eurasian element. The Eurasian element is further sub-divided into six components: East Asian taxa (with 30 species), European-Pakistani taxa (13), Himalayan taxa (11), Indian taxa (five), Eurasian taxa (six) and endemic (42; 13%). Additionally, 23 taxa are palaeotropical, nine are East Asiatic-North American, 14 are pantropical and 43 are cosmopolitan in distribution. There is a possibility that some of the endemic species are synonyms (Higuchi and Nishimura 2003; Gruber and Peer 2012).

The earliest mentions of *Plagiothecium* Schimp. in Pakistan go back to the 19^th^ century, when Brotherus (1898a, b) first reported *P.denticulatum* (Hedw.) Schimp. and *P.nemorale* (Mitt.) A.Jaeger. Later, Noguchi (1954) again reported *P.nemorale* from Pakistan. Karczmarz (1980) reported *Plagiotheciumcavifolium* (Brid.) Z.Iwats. from the Indian-administrated area of Kashmir. Subsequent studies (Higuchi 1992; Nishimura et al. 1993a, b) brought several new discoveries, thus increasing the number of species described in the genus. Thus, at the turn of the 20^th^ and 21^st^ centuries, four *Plagiothecium* species were recorded in Pakistan: *P.cavifolium*, *P.denticulatum*, *P.latebricola* Schimp. and *P.nemorale* (Higuchi and Nishimura 2003). These species are included in the checklist of *Plagiothecium* in Eurasia (Wolski et al. 2021).

The current initiative is to update the moss flora of Pakistan, by taxonomically revising the dominant families and the addition of new records to the moss flora of Pakistan. This self-funded project was started by Mr. Aamir Shehzad Khan, after his master’s work on the Bryopsida in Pakistan (Khan 2019; Khan et al. 2020; Khan et al. 2021; Koponen et al. 2022).

However, comparing these data with data from other countries in this region (e.g., Iran and India [nine species], or China [20 taxa]) we can see that the *Plagiothecium* flora of this country is extremely poor (Wolski et al. 2021). Taking these facts into account, research was undertaken to revise the *Plagiothecium* specimens available from Pakistan.

## ﻿Materials and methods

The current study is based exclusively on the Pakistani material, on the specimens deposited at the herbarium of Botanical Sciences Division, Pakistan Museum of Natural History (herbarium PMNH). This collection was made by Japanese bryologists during their Cryptogamic Expedition in Pakistan, which was organized through the collaboration of the National Science Museum (National Museum of Nature and Science), Tokyo, Japan (herbarium TNS) and the Pakistan Museum of Natural History (Higuchi 1992; Nishimura et al. 1993b). All the studied herbarium specimens were collected from the Nanga Parbat base camp and Mazeno base camp in 1990. The studied specimens have also been deposited at the herbarium of University of Lodz (LOD), Poland.

In addition, the investigated herbarium collection was supplemented by field research carried out by the second author from August 2018 to July 2020 in the northern areas of Pakistan, including Murree (Punjab Province), Galiyat-region, Swat Valley, Lower Dir (Khyber Pakhtunkhwa Province), and some selected areas of Azad Jammu and Kashmir (Pakistan).

Measurements were made in accordance with the methodology proposed by Wolski (2017, 2019). Both the qualitative and quantitative characteristics of the gametophyte were studied. Measurements were obtained from leaves that were torn off from the central part of the stem. Leaf cells were measured in all leaf zones, randomly selecting 30 cells from each leaf zone. Furthermore, a cross-section of the stem was made to measure the width of epidermal and parenchymal cells. Results were summarized, giving the minimum, maximum and average (M) value for each feature – these data were used to describe individual taxa.

The features of two similar specimens – later named Plagiotheciumhiguchiivar.higuchii and Plagiotheciumhiguchiivar.brevicellum – were statistically compared to see whether the differences are significant. However, due to the lack of normality in the distributions of individual variables, a non-parametric U-Mann-Whitney test was performed.

## ﻿Results and discussion

The conducted revision shows that the entire analyzed material is not very diverse. Most of the tested specimens belong to the *Plagiotheciumdenticulatum* complex. Thus, *P.cavifolium*, *P.latebricola* and *P.nemorale*, previously reported from this area, were not identified in the samples surveyed. The *P.denticulatum* complex dominated the examined material, within which the largest number of samples was represented by P.denticulatumvar.denticulatum, the remaining specimens belonged to a new taxon, not previously recorded from Pakistan, P.denticulatumvar.obtusifolium (Turner) Moore (*M. Higuchi 20499*).

### ﻿Taxonomic treatment

#### ﻿ Plagiothecium denticulatum

Taxon classificationPlantaeHypnalesPlagiotheciaceae

(Hedw.) Schimp.

E16791D4-8D03-5792-A48F-D95B2F9B9542

Plagiothecium denticulatum (Hedw.) Schimp., Bryologia Europaea 5: 190, 501, Tab. VIII (1851).
≡ Hypnum denticulatum Hedw., Species Muscorum Frondosorum 237 (1801). **Lectotype** (fide Ireland 1969): “St. Cr. 4. p. t. 30”, leg. Starke, Germany?, G (*n.v*.).

##### Description.

**Figure 1.**
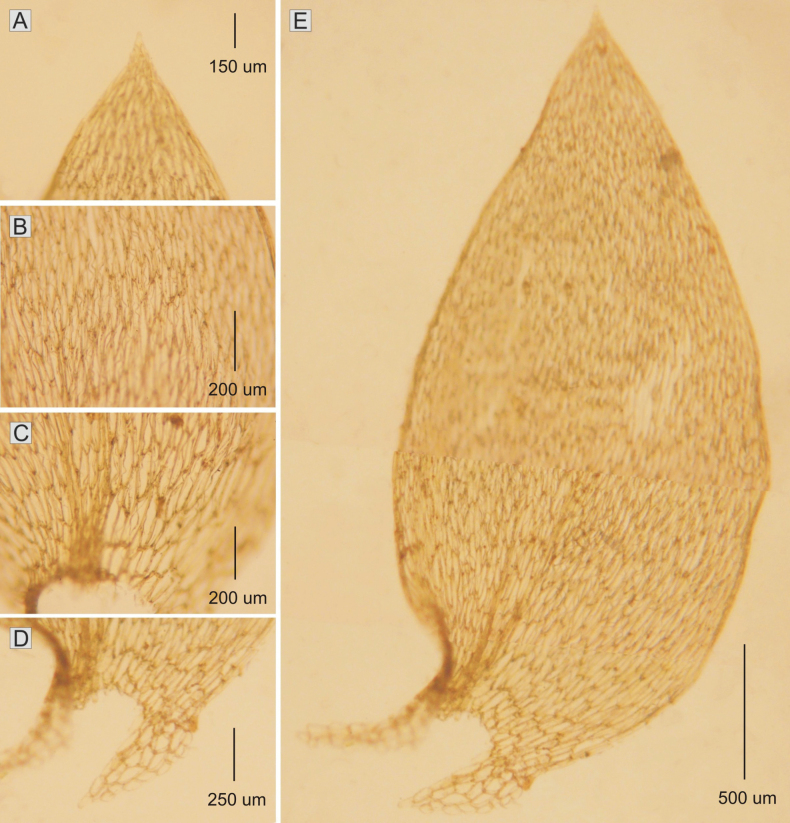
The most important taxonomic features of *Plagiotheciumdenticulatum***A** shape of apex **B, C** dimension of cells (**B** from the middle **C** basal part of the leaf) **D** decurrent cells **E** leaf shape (from *M. Higuchi 20462*).

Plants green to yellowish green, with metallic luster; stem in cross section rounded, 200–250 μm in diameter; leaves not folded, julaceous, elliptical-ovate, asymmetrical; leaves 2.2–3.0 × 1.0–1.3 (M 2.6 × 1.1) mm, shortly tapering to acute, denticulate apex; costae two, extending usually to ½ of the leaf length, ranging 440–810 (M 625) μm; cell areolation loose; length and width of cells very variable, but dependent on location: 85–140 × 15–28 (M 113 × 22) μm at the apex; 120–210 × 15–30 (M 165 × 23) μm at midleaf; 116–205 × 18–37 (M 160 × 28) μm toward insertion; broad decurrency 400–700 (M 555) μm; alar cells rounded, clearly inflated, 45–90 × 20–50 (M 68 × 35) μm; sporophytes not found in this material (Fig. 1). In these studies, *Plagiotheciumdenticulatum* has been recorded on soil and boulders.

#### ﻿ Plagiothecium denticulatum var. obtusifolium

Taxon classificationPlantaeHypnalesPlagiotheciaceae

(Turner) Moore

27ED5B4D-F6F5-5939-939B-20157669594B

**Figure 2.**
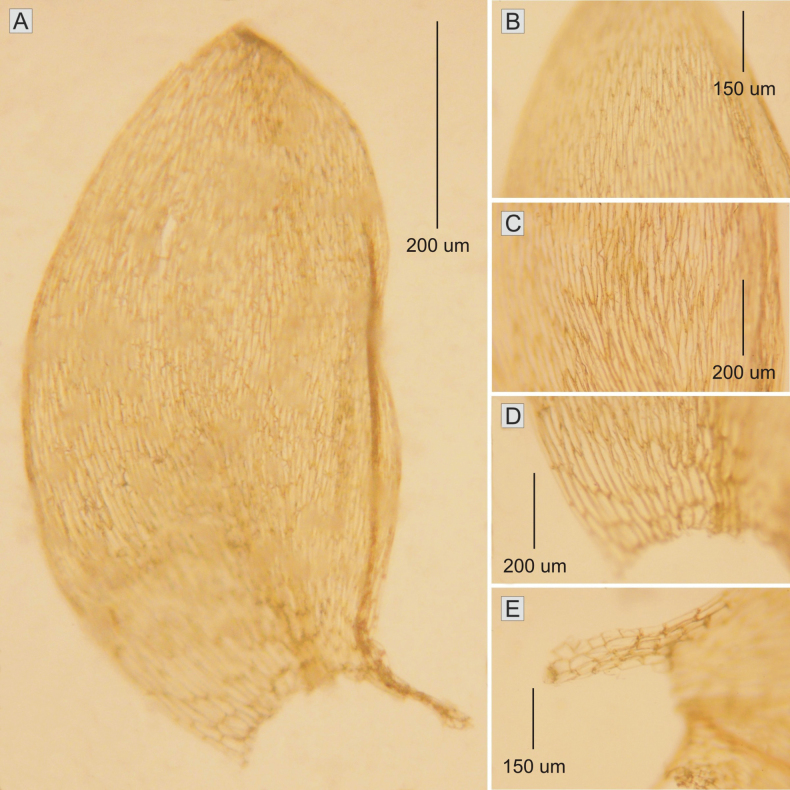
The most important taxonomic features of Plagiotheciumdenticulatumvar.obtusifolium**A** leaf shape **B–D** dimension of cells (**B** from the apex **C** the middle **D** basal part of the leaf) **E** decurrent cells (from *M. Higuchi 20499*).

**Figure 3.**
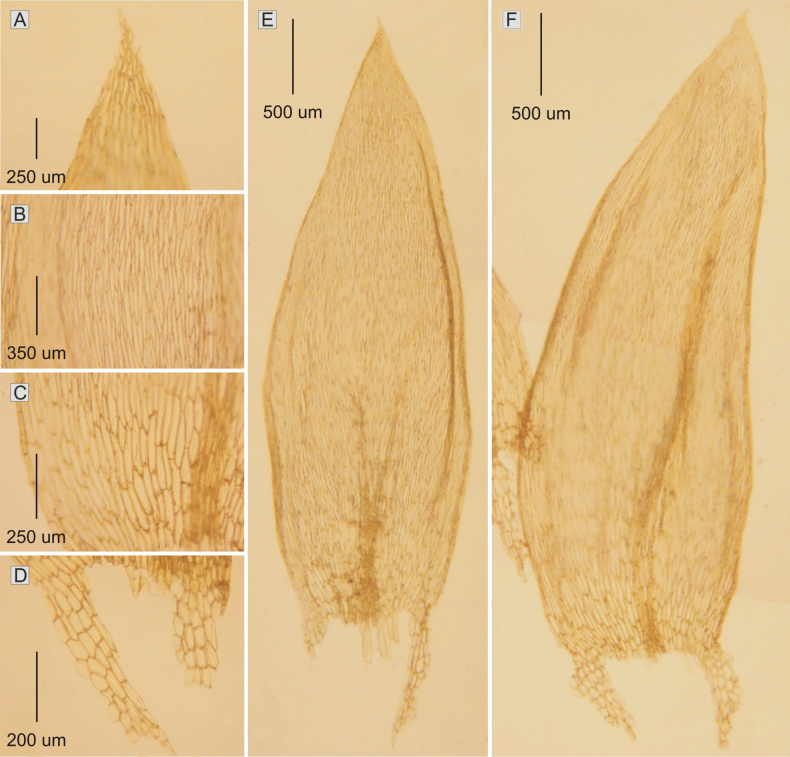
The most important taxonomic features of Plagiotheciumhiguchiivar.higuchii**A** apex serration **B, C** dimension of cells (**B** from the middle **C** basal part of the leaf) **D** decurrent cells **E, F** leaf shape (from *M. Higuchi 20460*).

Plagiothecium denticulatum var. obtusifolium (Turner) Moore, Proceedings of the Royal Irish Academy 1: 424 (1873).
≡ Hypnum denticulatum var. obtusifolium Turner, Muscologiae Hibernicae Spicilegium 146, T. 12, f. 2 (1804).
≡ Plagiothecium obtusifolium (Turner) J.J.Amann, Mémoire de la Société Vaudoise des Sciences Naturelles 3: 61 (1928). ***Holotype***: fig. 2, tabela 12 “T. 12, f. 2” (Turner 1804: 237) (fig. 3). **Epitype** (selected by Wolski et al. 2022) [Ireland], in summo montis Bulbein jugo, ab oculatissimo *D. Brown* lectam, benigne communicavit D. Templeton, BM000890810! (fig. 10).
= Plagiothecium sandbergii Renauld & Cardot, Contributions from the United States National Herbarium, 3: 274 (1895), **Lectotype** (selected by Wolski et al. 2022): U.S.A., Idaho, Kootenai County, Hope, *J.H*. Sandberg, *D.T*. Macdougal, *A.A*. *Heller 1174*, August 1892 (PC0132604!); ***isolectotypes***: NY507114!, available online; US70396!, available online; FH220148. Additional original material from *locus classicus* (not signed “No. 1174”): 456 NY507115!, available online. Additional Sandberg material, potentially from *locus* 457 *classicus*: PC0132605! Additional Sandberg material: FH220147.
= Plagiothecium denticulatum var. auritum Kern, Jahresbericht der Schlesischen Gesellschaft für Vaterländische Cultur 91(Abt. 2b): 97 (1914). ***Lectotype*** (selected by Wolski et al. 2022): [Italy] South Tirol, Ortler, Martelltal, in Felshöhlungen oberhalb der Cevedalehütte, *F. Kern s.n.*, 2350 m, 30 July 1913, herb. *I. Thériot* (PC0132639!).

##### Description.

Plants light green, with metallic luster; stem in cross section rounded, 150–200 μm in diameter; the central strand well developed, epidermal cells 12–20 × 15–31 (M 16 × 23) μm, the parenchyma thin-walled, 25–43 × 21–39 (M 34 × 30) μm; leaves very concave, not folded, julaceous, elliptical-ovate, very slightly asymmetrical, 1.6–1.7 × 0.8–0.95 (M 1.65 × 0.87) mm; the apex obtusely-apiculate; costae two, extending usually up to 1/3 or ½ of the leaf length, ranging 250–530 (M 390) μm; cell areolation loose; length and width of cells very variable, but dependent on location: 85–140 × 15–18 (M 113 × 17) μm at the apex; 95–170 × 12–18 (M 133 × 15) μm at midleaf; 98–200 × 15–25 (M 150 × 20) μm toward insertion; broad decurrency 260–400 (M 330) μm; alar cells rounded-rectangular, 51–98 × 21–31 (M 75 × 26) μm; sporophytes not found in this material (Fig. 2). In these studies, Plagiotheciumdenticulatumvar.obtusifolium (*M. Higuchi 20499*) has been recorded on soil.

The next two samples (*M. Higuchi 20460* and *M. Higuchi 20479*) were characterized by a unique combination of qualitative and quantitative features of their gametophytes. They did not match any of the taxa of the genus currently known in the Northern and Southern Hemispheres.

The specimen collected by M. Higuchi (*M. Higuchi 20460*) represents two morphotypes that are microscopically very different from each other. This material differs in the length and width of the leaf, the shape of the apex, the length and width of the cells of the top, middle and basal part of the leaf – which are one of the most important taxonomic features for the whole genus. In addition, the result of the U Mann-Whitney test showed that the length and width of the cells of individual parts of the leaf of these morphotypes differ statistically significantly (p<0.001) from each other. Thus, it was proposed to recognise two varieties – Plagiotheciumhiguchiivar.higuchii and P.higuchiivar.brevicellum.

#### ﻿ Plagiothecium higuchii

Taxon classificationPlantaeHypnalesPlagiotheciaceae

G.J.Wolski sp. nov.

9E5C90C2-080E-5553-B55E-149E88C59D46

##### Type.

Pakistan, Mt. Nanga Parbat, Mazeno Base Camp, 4000 m alt, on soil, 15 September 1990, *M. Higuchi 20460*, ***holotype*** LOD15016, ***isotype***PMNH.

##### Description.

Plants green-yellow to golden-gold, without metallic luster; stems complanate-foliate, 3.0–4.0 cm long, in cross-section rounded, with a diameter of 290–360 (M 325) μm, the central strand well developed, epidermal cells 11–22 × 13–32 (M 16 × 22) μm, the parenchyma thin-walled, 26–50 × 23–45 (M 38 × 34) μm; leaves quite loosely arranged on the stem, concave, symmetrical to asymmetrical, ovate, those leaves from the middle of the stem 3.4–4.3 × 1.2–1.9 (M 3.8 × 1.5) mm; the apex acuminate and denticulate; costae two, thick and strong, usually to 1/2 of the leaf length, reaching 0.5–1.2 mm; laminal cells symmetrical, in unregulated transverse rows, the length and width very variable, but dependent on location: 107–250 × 16–24 (M 178 × 20) μm at apex, 139–266 × 21–33 (M 203 × 27) μm at mid-leaf and 139–266 × 21–33 (M 203 × 27) μm towards insertion, cell areolation very loose; decurrency long, 300–500 (M 400) μm, composed of 4–6 rows of rectangular and spherically inflated cells, 48–144 × 26–70 (M 96 × 48) μm. Sporophytes so far unknown (Fig. 3). In these studies, Plagiotheciumhiguchiivar.higuchii (*M. Higuchi 20460*) has been recorded on soil.

##### Etymology.

The present species is named in honor of Professor Masanobu Higuchi, who participated in the Studies on Cryptogams in the Western Himalayas in Pakistan project, and who collected the specimen (*Higuchi 20460*) chosen here as the holotype of *Plagiotheciumhiguchii*.

#### ﻿ Plagiothecium higuchii var. brevicellum

Taxon classificationPlantaeHypnalesPlagiotheciaceae

G.J.Wolski var. nov.

7D653D82-E16C-5899-BDB1-00F4AB9B69E7

##### Type.

Pakistan, Mt. Nanga Parbat, Mazeno Base Camp, 4000 m alt, on soil, 15 September 1990, *M. Higuchi 20460pp*, ***holotype***LOD 15017, ***isotype***PMNH.

##### Description.

**Figure 4.**
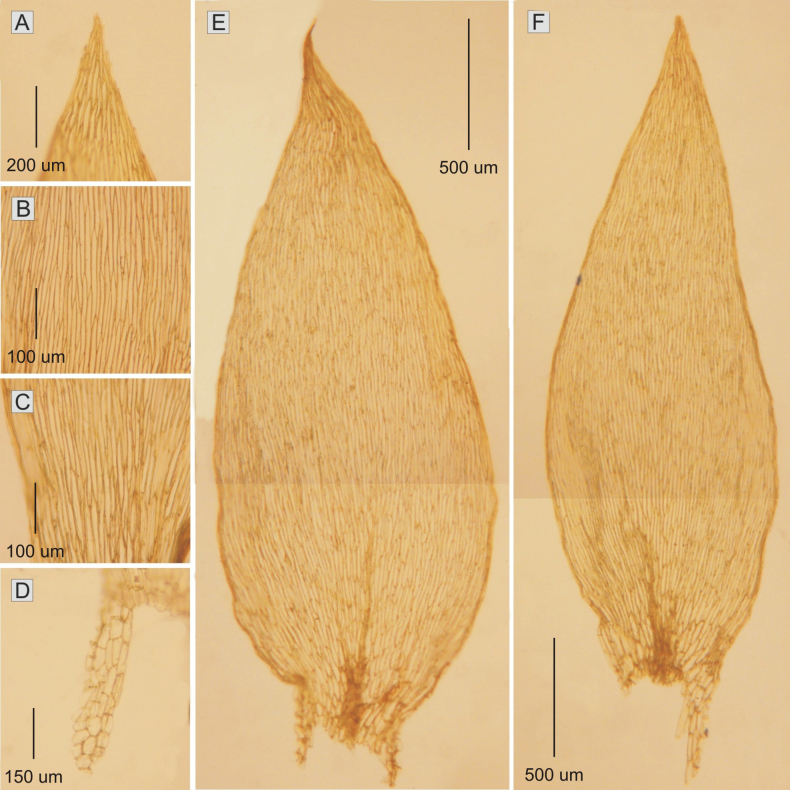
The most important taxonomic features of Plagiotheciumhiguchiivar.brevicellum**A** apex serration **B, C** dimension of cells (**B** from the middle **C** basal part of the leaf) **D** decurrent cells **E, F** leaf shape (from *M. Higuchi 20460*).

Plants green-yellow to golden-gold, without metallic luster. Stems complanate-foliate, 3.0–4.0 cm long, in cross-section rounded, with a diameter of 310–388 μm, epidermal cells 10–16 × 21–33 (M 13 × 27) μm, the parenchyma thin-walled, 14–58 × 18–51 (M 34 × 35) μm; leaves quite loosely arranged on the stem, concave, symmetrical to gently asymmetrical, ovate, those leaves from the middle of the stem 2.9–3.2 × 1.0–1.2 (M 3.1 × 1.1) mm; the apex acute and apiculate, denticulate; costae two, thick and strong, usually to 1/2 of the leaf length, reaching 0.5–1.5 (M 1.0) mm; laminal cells symmetrical to slightly asymmetrical, the length and width very variable, but dependent on location: 70–170 × 10–19 (M 120 × 15) μm at apex, 100–200 × 14–26 (M 150 × 20) μm at mid-leaf and 100–200 × 19–36 (M 150 × 28) μm towards insertion, cell areolation loose; decurrency long, 390–650 (M 520) μm, composed of 4–6 rows of rectangular and spherically inflated cells, 36–116 × 18–67 (M 76 × 43) μm. Sporophytes unknown so far (Fig. 4). In these studies Plagiotheciumhiguchiivar.brevicellum (*M. Higuchi 20460*) has been recorded on soil.

##### Etymology.

The variety name *brevicellum* refers to breve- [Lat.] short; -cellus [Lat.] cells and indicates the most distinctive features between varieties – leaves with short cells.

#### ﻿ Plagiothecium filifolium

Taxon classificationPlantaeHypnalesPlagiotheciaceae

G.J.Wolski sp. nov.

871A9D4F-4CC6-53CD-86B7-3808D499E79B

##### Type.

Pakistan, Mt. Nanga Parbat, Mazeno Base Camp, 4000 m alt, on boulder, 15 September 1990, *M. Higuchi 20479*, ***holotype***LOD 15018, ***isotype***PMNH.

##### Description.

**Figure 5.**
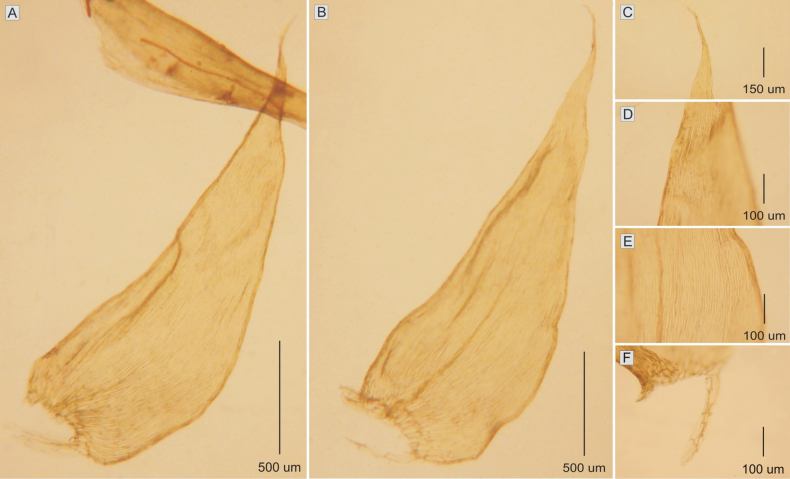
The most important taxonomic features of *Plagiotheciumfilifolium***A, B** leaf shape **C** leaf apex **D** folding of the apex **E, F** dimension of cells (**E** from the middle **F** basal part of the leaf) (from *M. Higuchi 20479*).

Plants green-yellow, without metallic luster; stems complanate-foliate, 1.0–1.5 cm long; cross-section rounded, with a diameter of 200–300 μm, the central strand well developed, epidermal cells 9–13 × 11–15 (M 11 × 13) μm, the parenchyma thin-walled, 15–21 × 16–28 (M 18 × 22) μm; leaves folded, most strongly in the upper part of leaves, concave, asymmetrical, those leaves from the middle of the stem 1.6–2.2 × 0.5–0.8 (M 1.9 × 0.66) mm; leaves gradually tapering to long, acuminate, not denticulate apex; costae two, very thin and delicate, extending up to 1/2 of the leaf length, reaching 140–423 (M 282) μm; laminal cells rather symmetrical, the length and width very variable, but dependent on location: 97–160 × 7–10 (M 127 × 8) μm at apex, 105–180 × 7–9 (M 150 × 8) μm at mid-leaf and 70–165 × 8–11 (M 118 × 9) μm towards insertion, cell areolation tight; decurrency short, 220–530 μm, composed of 3 rows of gently inflated, rectangular cells, 47–147 × 12–21 (M 97 × 16) μm. Sporophytes unknown so far (Fig. 5). In these studies, *Plagiotheciumfilifolium* (*M. Higuchi 20479*) has been recorded on boulders.

##### Etymology.

Filum- [Lat.] thread; -folium [Lat.] leaf. The present species is named in reference to the most distinctive feature – its threadlike leaf apex.

### ﻿Discussion

Previously, only four species had been reported from Pakistan: *Plagiotheciumcavifolium*, *P.denticulatum*, *P.latebricola* and *P.nemorale* (Brotherus 1898a, b; Noguchi 1954; Karczmarz 1980; Higuchi 1992; Nishimura et al. 1993b; Higuchi and Nishimura 2003). Noguchi (1954) also reported *P.sylvaticum* (Brid.) Bruch and Schimp., but this is now considered to be a synonym of *P.nemorale* (Wolski et al. 2022). The current research revealed five taxa belonging to this genus, including three new to science and one new to Pakistan. Thus, the conducted research added another four *Plagiothecium* taxa to the known flora of Pakistan.

Compared to neighboring countries, the four species previously reported from Pakistan (*Plagiotheciumcavifolium*, *P.denticulatum*, *P.latebricola* and *P.nemorale*) were considered a very low number (Wolski et al. 2021). Of course, the higher numbers of species of *Plagiothecium* recorded from China (20 taxa) (e.g. He and Redfearn 1995; Redfearn et al. 1996; Wynns 2015; Zuo et al. 2011; Wolski, Nowicka-Krawczyk 2020) or India (nine taxa) (e.g. Gangulee 1980; Dandotiya et al. 2011; Asthan and Sahu 2015; Wolski and Nowicka-Krawczyk 2020) result from the larger size of these countries, greater diversity of habitats, but also from a longer history of bryological research. On the other hand, ten taxa have been reported from Iran (Wolski et al. 2021), which has a similar climate and habitats to Pakistan.

As indicated by Wolski et al. (2021) in the Northern Hemisphere, the *Plagiotheciumdenticulatum* is the most frequently recorded. This was also the case in the above studies. Most of the specimens studied represented this species. It is also not surprising to find Plagiotheciumdenticulatumvar.obtusifolium, which is new to Pakistan, in the examined material, not only because it is recorded in many neighboring countries, including China or Iran (Wolski et al. 2021), but also because it is a montane taxon (Wolski et al. 2022) and all specimens studied were collected between 3.430 m to 4.000 m.

This research showed that some of the specimens have unique combinations of qualitative and quantitative gametophytic features. A specimen with complanate-foliate stems; folded, concave, asymmetrical leaves gradually tapering to long, acuminate, not denticulate apex, with a decurrency composed of gently inflated, rectangular cells has been named *Plagiotheciumfilifolium*. There is no species in the Northern Hemisphere with this set of features. The most similar to this taxon is *P.latebricola*, which, however, is characterized by a symmetrical leaf, narrow decurrency with non-inflated cells (e.g. Nyholm 1965; Lewinsky 1974; Wolski et al. 2021). *Plagiotheciumfilifolium* is also different from *Plagiotheciumsubulatum*, recently transferred to *Vesicularia* (Müll. Hal.) Müll. Hal. (Müller and Wynns 2020) and now known as *Vesiculariasubulata* (Broth.) J.T. Wynns & Frank Müll. The aforementioned can be characterized as medium-sized plants, complanate or fluffy, leaves weakly overlapping, flat, short, broad, ovate, asymmetric, with erect margins and gemmae produced in dense clusters at leaf tips (Wolski et al. 2021).

Specimens with leaves quite loosely arranged on the stem, concave, symmetrical to asymmetrical, ovate, with a denticulate apex, loose cell areolation and wide decurrency, composed of rectangular and spherically inflated cells have been named *Plagiotheciumhiguchii*. These specimens also represent a unique set of features hitherto unknown in the Northern or Southern Hemispheres (Ireland 1986, 1992, 2001; Buck and Ireland 1989; Buck 1998; Wolski et al. 2021). Decurrencies, composed of spherically inflated cells, asymmetric, concave leaf or the dimensions of the cells distinguish *P.higuchii* from the common Northern Hemisphere *P.nemorale* (Wolski 2020; Wolski and Nowicka-Krawczyk 2020). On the other hand, this feature indicates a similarity with representatives of PlagiotheciumsectionPlagiothecium, because taxa from this section usually have a wide decurrency with a group of cells more or less clearly inflated (Wynns et al. 2018).

The taxa from the Northern Hemisphere most similar to *P.higuchii* would be: *P.denticulatum*, *P.platyphyllum* Mönk. or *P.ruthei* Limpr., however, they differ in the arrangement of the leaves on the stem, in the shape and dimensions of the leaf, and the dimensions of the leaf cells, which shows the striking distinctiveness of the examined material against the previously described taxa (Ireland 1986, 1992; Buck 1998; Wolski 2020; Wolski and Nowicka-Krawczyk 2020; Wolski et al. 2021).

As studies of *Plagiothecium* indicate (e.g. Ireland 1969; Iwatsuki 1970; Lewinsky 1974; Wynns et al. 2018; Wolski and Nowicka-Krawczyk 2020) the dimensions of the leaf cells are one of the most important taxonomic features of these plants. The conducted research, confirmed by statistical analyses, shows that the material of *P.higuchii* represents two different morphotypes, therefore two varieties were proposed within this species, Plagiotheciumhiguchiivar.higuchii, with long leaf cells, and P.higuchiivar.brevicellum, with short leaf cells.

The present study describes three taxa, Plagiotheciumhiguchiivar.higuchii, P.higuchiivar.brevicellum, and *P.filifolium*, as new to science and reports another taxon, P.denticulatumvar.obtusifolium, as new to Pakistan. With the four species reported earlier this brings the number of recognized species and infraspecific taxa of *Plagiothecium* in Pakistan to eight.

**Figure 6.**
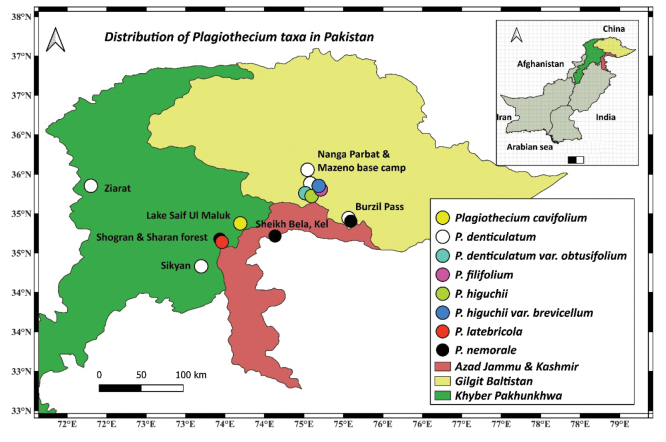
A distribution map showing all the currently known localities of *Plagiothecium* species in Pakistan.

On the basis of herbarium specimens and literature analysis, it was possible to determine the range of the described species of the genus in Pakistan:

- *Plagiotheciumcavifolium* has been recorded so far from: Khyber Pakhtunkhwa, Kaghan Valley, Lake Saif Ul Maluk, 3150 m (*Higuchi 19888*, *Higuchi 19891*, Higuchi 1992) (Fig. 6).
- *Plagiotheciumdenticulatum* has been recorded so far from: Khyber Pakhtunkhwa, Chitral, Ziarat, 7400 m and Gilgit-Baltistan, Minimarg, Burzil Pass, 10 000 to 11 000 m (Brotherus 1898a, b); Gilgit-Baltistan, Nanga Parbat Base camp 3430 m, and Mazeno Base camp, 4000 m (*Higuchi 20462*, *20475*, Higuchi 1992); Khyber Pakhtunkhwa, Mansehra, Sikyan near Nadi, 2020 m (Nishimura et al. 1993b) and Nanga Parbat Base Camp and Nanga Parbat Mazeno Base Camp, at 3430–4000 m (*M. Higuchi 20462*; *M. Higuchi 20464*; *M. Higuchi 20479*; *M. Higuchi 20510*) (Fig. 6).
- *Plagiotheciumdenticulatum* var. *obtusifolium* has been recorded so far from: Gilgit-Baltistan, Nanga Parbat, Base Camp, at 3430 m (*M. Higuchi 20499*) (Fig. 6).
- *Plagiotheciumfilifolium* has been recorded so far from: Gilgit-Baltistan, Nanga Parbat, Mazeno Base Camp, at 4000 m (*M. Higuchi 20479*) (Fig. 6).
- *Plagiotheciumhiguchii* var. *higuchii* has been recorded so far from: Gilgit-Baltistan, Nanga Parbat, Mazeno Base Camp, at 4000 m (*M. Higuchi 20460*) (Fig. 6).
- *Plagiotheciumhiguchii* var. *brevicellum* has been recorded so far from: Gilgit-Baltistan, Nanga Parbat, Mazeno Base Camp, at 4000 m (*M. Higuchi 20460*) (Fig. 6).
- *Plagiotheciumlatebricola* has been recorded so far from: Khyber Pakhtunkhwa, Mansehra, Shogran – Sali Hut, 2710 m (Nishimura et al. 1993b) (Fig. 6).
- *Plagiotheciumnemorale* has been recorded so far from: Gilgit-Baltistan, Minimarg, Burzil Pass, 9000 to 10 000 m and 10 000 to 11 000 m (Brotherus 1898a); Azad Jammu and Kashmir, Shekh Bela, between Shardi and Kel, alt. 2000–2100 m (Noguchi 1954) (as *P.sylvaticum*); Khyber Pakhtunkhwa, Mansehra, Sharan Forest, 2400 m (Nishimura et al. 1993b) (Fig. 6).

### ﻿A key to the species of the genus *Plagiothecium* from Pakistan

**Table:** 

1	Angular cells of decurrency rounded, inflated, forming distinct auricles	**2**
–	Angular cells of decurrency rectangular, not inflated, not forming distinct auricles	**5**
2	Leaves asymmetrical; apex acute and denticulate	***P.denticulatum***
–	Leaves symmetrical or slightly asymmetric; apex denticulate or not	**3**
3	Plants with acuminate, denticulate apex	**4**
–	Plants with obtuse, not denticulate apex	**P.denticulatumvar.obtusifolium**
4	Cells from the middle part of the leaves long and wide (139–266 × 21–33)	**P.higuchiivar.higuchii**
–	Cells from the middle part of the leaves shorter and narrower (100–200 × 14–26 μm)	**P.higuchiivar.brevicellum**
5	Leaves asymmetric and folded	***P.filifolium***
–	Leaves symmetric and not folded	**6**
6	Cells from the middle part of the leaves < than 10 μm wide	***P.latebricola***
–	Cells from the middle part of the leaves > than 10 μm wide	**7**
7	Stem julaceus; leaves strongly concave; apex not denticulate	***P.cavifolium***
–	Stem not julaceus; leaves rather flat; apex denticulate	***P.nemorale***

## Supplementary Material

XML Treatment for Plagiothecium denticulatum

XML Treatment for Plagiothecium denticulatum var. obtusifolium

XML Treatment for Plagiothecium higuchii

XML Treatment for Plagiothecium higuchii var. brevicellum

XML Treatment for Plagiothecium filifolium

## ﻿Additional information

### Author ORCIDs

Grzegorz J. Wolski https://orcid.org/0000-0003-1480-8003

Aamir Shehzad Khan https://orcid.org/0000-0002-9039-2524

Beata Paszko https://orcid.org/0000-0003-3044-1766

### Data availability

All of the data that support the findings of this study are available in the main text or Supplementary Information.

## Supplementary materials

Supplementary material 1 Raw data of new taxa from Pakistan Data type xlsx Explanation note The raw data contains two sheets, with morphological data and statistical test results. https://binary.pensoft.net/file/941797This dataset is made available under the Open Database License (http://opendatacommons.org/licenses/odbl/1.0/). The Open Database License (ODbL) is a license agreement intended to allow users to freely share, modify, and use this Dataset while maintaining this same freedom for others, provided that the original source and author(s) are credited.Grzegorz J. Wolski, Aamir Shehzad Khan, Beata Paszko
